# Athletic performance levels across biological maturity status in youth soccer players

**DOI:** 10.3389/fspor.2026.1856821

**Published:** 2026-06-22

**Authors:** Giuseppe Scardina, Guglielmo Pillitteri, Domenico Savio Salvatore Vicari, Alberto Canzone, Filipe Manuel Clemente, Valerio Giustino, Antonino Bianco

**Affiliations:** 1 Sport and Exercise Sciences Research Unit, Department of Psychology, Educational Science and Human Movement, University of Palermo, Palermo, Italy; 2 Department of Neurosciences, Biomedicine and Movement Sciences, University of Verona, Verona, Italy; 3 Escola Superior Desporto e Lazer, Instituto Politécnico deViana do Castelo, Viana do Castelo, Portugal; 4 Sport Physical Activity and Health Research & Innovation Center, Applied Research Institute, Polytechnic University of Coimbra, Coimbra, Portugal; 5 Gdansk University of Physical Education and Sport, Gdansk, Poland

**Keywords:** biological maturation, football, maturation, maturity offset, peak height velocity, performance, soccer, talent identification

## Abstract

**Introduction:**

Biological maturation can produce substantial inter-individual variability in physical performance among youth soccer players. However, whether its influence differs in magnitude across physical domains has been little examined within a testing battery.

**Methods:**

Forty-four youth male soccer players (13.12 ± 1.75 years; U13-U16) were assessed for countermovement jump (CMJ) height, lower limb peak power, sprint speed over 10 and 30 m (reflecting acceleration and maximal velocity, respectively), and VO2max. Predicted maturity status was classified into pre-, circa-, and post-PHV (peak height velocity) groups using the modified Mirwald equation. Group differences were analyzed using the one-way ANOVA.

**Results:**

Post-PHV group outperformed both pre- and circa-PHV groups across all outcomes (*p* ≤ 0.05). Effect sizes were large in all outcomes, and the most significant were recorded for lower limb peak power (*ε*2 = 0.73), followed by 30-m sprint (*η*2 = 0.63), 10-m sprint (*η*2 = 0.51), VO2max (*ε*2 = 0.51), and CMJ height (*η*2 = 0.40).

**Conclusion:**

Maturity status is associated with large performance advantages across all tested domains in youth soccer players. Post-PHV players demonstrated significantly higher performance in jumping, sprinting, aerobic capacity, and lower limb peak power and support the integration of predicted maturity assessments in physical profiling, training program design, as well as for facilitating talent identification.

## 1 Introduction

Soccer is an intermittent high-intensity team sport that requires a complex interplay of physical, technical, and tactical skills (1, 2). Players are required to perform repeated sprints, jumps, rapid changes of directions, and sustained aerobic activity throughout a match lasting 90 min or more (1). The multiple physical demands of soccer place emphasis on characteristics such as explosive power, speed, and aerobic endurance, which are key determinants of match performance and player success (2, 3). Adolescence is characterized by inter-individual heterogeneity in somatic development, with variations in height, body mass, and muscle mass observed among peers of the same chronological age (4). This heterogeneity is governed by biological maturation, which shows considerable variation in both timing and tempo across individuals (5). A widely used reference for characterizing this process is the peak height velocity (PHV), defined as the achievement of maximum height growth velocity during adolescence (6). Maturity-associated variation has been extensively documented in the literature (7, 8). Youth who show an advanced biological maturation demonstrate greater skeletal muscle mass, enhanced neuromuscular function, elevated anabolic hormone concentrations, particularly testosterone, growth hormone, insulin-like growth factor-1, and superior cardiopulmonary capacity compared with less mature peers (7, 8). These physiological differences translate into meaningful and well-documented physical performance advantages, including sprint speed (0.2–0.6 s over 10–30 m), greater vertical jump height (differences of 5–10 cm), higher lower-limb peak power output (exceeding 1,000 W between extreme PHV groups), and higher maximal aerobic capacity (VO2max differences of 5–10 mL/kg/min), all of which are key determinants of success in youth soccer (7–10). Critically, players of the same chronological age can differ significantly in maturity status, meaning that age-group comparisons frequently conflate maturity effects with true inter-individual differences in physical ability (9).

Several methods have been proposed to estimate maturity status in applied settings. Skeletal age assessment via hand-wrist radiography is considered the gold standard but is costly and raises ethical concerns regarding radiation exposure (9). Among non-invasive field-based alternatives, two methods are most widely used in youth soccer, that is the Khamis-Roche (KR) method and the modified Mirwald equation. The KR method predicts the percentage of predicted adult height, and it requires parental height data, which may not always be available (e.g., lack of parental consent, adopted players) (11). The modified Mirwald equation estimates the maturity offset (i.e., the number of years from PHV) and requires only players' anthropometric measurements, making it more practical in applied settings (12). It is important to note that this equation has an inherent prediction error of approximately 0.5–1 year, and this error is known to be greatest in the circa-PHV period, immediately around PHV, and it introduces the possibility that some players near the classification limits may be misclassified (12).

While the influence of biological maturation on physical performance is well established, its domain-specific magnitude, in relation to assessed performance characteristics, remains less clear. Gundersen et al. (2026) demonstrated that, in youth male soccer players, the association between maturity status and sprint and countermovement jump (CMJ) performance reached a plateau and began to diminish at relatively earlier maturity stages (approximately circa-PHV), while associations with maximal strength and aerobic capacity continued to strengthen into the post-PHV stage (13), suggesting that the maturity status influence different performance domains. Zghal et al. (2025) reported that, in youth soccer players, force-oriented capacities (maximal voluntary force, rate of force development) and velocity-oriented capacities (sprint velocity, jump height) develop at different rates across maturity stages, with force qualities improving more rapidly during the circa-PHV stage and velocity qualities showing greater relative gains earlier in maturation (14). Deprez et al. (2015) demonstrated that maturation-related differences in lower limb power were larger when assessed using squat jump (SJ) compared to CMJ, suggesting that the stretch-shortening cycle (SSC) contribution to jump performance introduces additional variability in the maturation-performance relationship (15). Together, these studies suggest that a testing battery is necessary to fully characterize the domain-specific nature of maturation-related differences in performance. The neuromuscular basis of these domain-specific differences lies in the development of the SSC and changes in muscle architecture maturation-related. During the pre-PHV stage, the SSC is limited by relatively underdeveloped muscle-tendon unit stiffness and short-range stiffness, the elastic energy storage and release mechanism that amplifies force production during rapid SSC such as in the CMJ and sprint push-off phase (16). As maturity status advances through the circa- and post-PHV stages, increasing testosterone and IGF-1 concentrations lead to an increase in pennation angle, muscle fiber cross-sectional area, and muscle-tendon unit stiffness, improving both the capacity to store elastic energy and the rate at which it can be released (16).

Few studies have assessed a testing battery including field-based tests within cohorts classified by predicted PHV, limiting comparisons across performance domains. Therefore, this study examined maturity status differences across multiple performance measures in youth male soccer players. It was hypothesized that post-PHV players would outperform pre- and circa-PHV peers across all performance outcomes, and that the magnitude of these maturity status differences would be non-uniform across performance domains, reflecting the differential hormonal and neuromuscular adaptations associated with advancing maturation.

## 2 Materials and methods

### 2.1 Study design

This research was designed as a cross-sectional study with the aim of evaluating any differences in CMJ height, sprint speed, lower limbs power output, and maximal oxygen uptake (VO_2_max), among youth male soccer players classified into groups based on their maturity status. The latter was determined by estimating the PHV using the modified Mirwald equation (12).

The study was approved by the Bioethics Committee of the University of Palermo (n. 307/2025 – prot. n. 60637-2025) and carried out in accordance with the principles of the Declaration of Helsinki.

### 2.2 Participants

The study included 44 youth male soccer players (mean age: 13.12 ± 1.75 years) from U16 (*n* = 11), U15 (*n* = 14), and U13 (*n* = 19) categories of a local soccer club. Eligibility criteria required that participants had 3 years of soccer experience and no injuries or medical conditions that could affect their physical performance at the time of testing or in the previous 6 months. Using the Participant Classification Framework proposed by McKay et al. (2022), the players in this study are classified as Tier 2/3 (developmental to competitive-regional level), reflecting their participation in organized regional youth soccer competition in Italy (17). For participant recruitment, a member of the Research Unit contacted the local soccer club to present the research. Subsequently, it was presented to the players and parents that had expressed their willingness to participate. Parents of the participants provided an informed written consent for study participation.

### 2.3 Measurements

Data collection was conducted during the regular training season at the soccer club's own facilities between March and April 2025 by the same investigators and in the same time slot (i.e., between 5:00 p.m. and 7:00 p.m.) in order to minimize time-of-day effect.

Prior to the assessment, all participants underwent a standardized 10-minute warm-up protocol, supervised by the team's athletic trainer. This warm-up followed the established Raise, Activate, Mobilize, and Potentiate (RAMP) framework (18).

The tests were administered in the following block order for all participants: CMJ, lower limbs power output, sprint speed over distances of 10 and 30 meters, and maximal oxygen uptake (VO_2_max).

Participants were asked to perform each test with maximum effort to ensure reliable data collection. Although participants had already taken these tests at least once during their athletic career, detailed procedural explanations were provided to ensure consistency across all testing sessions.

#### 2.3.1 Countermovement jump (CMJ) test

The CMJ test is designed to assess vertical jump height. In this test, participants began from a standing position inside an optical detection system (Optojump, Microgate; Bolzano, Italy), performed a rapid downward movement to a 90° knee angle, and then immediately jumped vertically as high as possible with their hands on their hips to minimize measurement errors (19). A standardized verbal cue “Jump as high as you can!” was delivered by the same investigator at the beginning of each trial. No feedback regarding jump height was provided between trials, to avoid performance anchoring effects. The Optojump system has demonstrated excellent validity for jump height measurement compared to force platform reference standards (ICC = 0.99; SEM < 0.5 cm) (20), and high test-retest reliability in youth athletic populations (CV < 2%) (21).

Participants performed 3 jump trials with 1-minute of passive recovery between them, and the best jump was used for statistical analysis. The jump height was measured in centimeters (cm). Peak power output during the CMJ was estimated using the Sayers equation (22), a validated regression formula that incorporates both vertical jump height and body mass to provide an estimate of lower limb peak power output, expressed in watts (W). This equation is as follows:

PeakPower(W)=(60.7×JumpHeight(cm))+(45.3×BodyMass(kg))−2055

#### 2.3.2 Sprint speed tests

The sprint speed tests evaluate maximum sprinting ability over short distances. Participants were asked to sprint from a standing position over a distance of 30 meters, and their sprint performance at 10 meters and 30 meters were recorded through an action camera (GoPro 9, GoPro Inc., San Mateo, CA, USA). To mark the distances over the sprint lane a cone was positioned at the start, at 10 meters and at 30 meters. The camera was set to a frame rate of 120 frames per second with an automatic shutter speed (minimum 1/500 s) to minimize motion blur during maximal sprint efforts. All video sequences were analyzed by the same investigator, who was blinded to each participant's maturity status, to control for potential assessor bias (23, 24). The camera was mounted on a tripod at a height of 1.0 m, at 15 m from the sprint lane and at a lateral distance of 10 m from the sprint lane, to minimize angular parallax error associated with off-axis filming. The 10 m distance was selected to assess early acceleration capacity, which is primarily force-oriented and sensitive to neuromuscular maturation, while the 30 m distance captures the transition to maximal sprint velocity (25). These distances align with the normative youth soccer literature and reflect the acceleration profiles most relevant to soccer-specific sprinting demands (10, 26). Video analysis was performed using Kinovea software (v. 0.9.5) following a two-dimensional spatial calibration against a known reference distance (a 10 m segment of marked track visible within the camera frame). Sprint times were defined as the interval between the participant's first forward movement and the moment the lead foot crossed each distance marker.

Participants performed 3 sprint trials with 2-minute of ­passive recovery between them, and the best 10-meter and 30-meter­ sprints were used for statistical analysis. The sprint performance was measured in seconds (s) (25).

#### 2.3.3 Maximal oxygen uptake (VO_2_max)

The VO_2_max was estimated using the Yo-Yo Intermittent Recovery test level 1 (YYIR1). This field test involves repeated 2 × 20-meter shuttle runs at progressively increasing speeds, cued by acoustic signals, with short recovery periods between running bouts. Each running bouts is followed by a 10-second of active recovery, during which participants jog 2 × 5 meters. All participants had prior experience with the YYIR1, as the test is routinely administered at the club, reducing familiarization effects. Standardized verbal instructions and a brief demonstration were provided before testing by the same investigator. Participants were instructed to refrain from vigorous exercise in the 24 h preceding testing. The acoustic signal was delivered via the validated YYIR1 audio file provided by the test's official protocol guidelines (27). Test termination criteria (two consecutive failures to reach the turning line within the signal) was standardized and uniformly applied by the same investigator.

It begins with 4 running bouts at a speed of 10–13 km/h (covering 0–160 m), followed by 7 running bouts at 13.5–14 km/h (covering 160–440 m). Thereafter, the test continues with incremental increases of 0.5 km/h in speed every 8 running bouts (i.e., after 760, 1,080, 1,400, 1720m, and so on) until exhaustion (27). The total distance covered was then used to estimate the VO_2_max, expressed in milliliters of oxygen per kilogram of body weight per minute (mL/kg/min) using the following equation:

$$VO_{2}max\;(ml/kg/min)=IR1\;distance\;(m)\times 0.0084+36.4$$

where IR stands for intermittent recovery (28).

#### 2.3.4 Maturity status assessment

The exact chronological age was calculated by determining the time interval between each participant's birth date and the test date. This calculation involved subtracting the birth date from the test date, with the resulting age expressed as a decimal value to the nearest 0.1 year. Anthropometric measurements, including height, weight, sitting height, and lower limb length were collected. Standing height was measured using a standard stadiometer (maximum height recordable: 220 cm; resolution: 1 mm). Participants stood without shoes, with heels together, arms at their sides, and shoulders relaxed (29). Body weight was measured using a Seca electronic scale (maximum weight recordable: 300 kg; resolution: 100 g; Seca, Hamburg, Germany). Participants stood without shoes and wearing underwear (29). Sitting height was measured with participants seated on a flat bench. Participants sat straight with their back against the wall, thighs fully supported on the bench, knees bent at 90 degrees, feet flat on the floor, and hands resting on their thighs. Sitting height was defined as the vertical distance from the sitting surface to the vertex for the head and length was calculated as the difference between standing height and sitting height (6). Maturity status was calculated using the modified Mirwald equation, which estimates the maturity offset, that is the number of years from PHV (12). The predictive equation is as follows:

MaturityOffset:−9.236+(0.0002708×LegLength×SittingHeight)+(−0.001663×Age×LegLength)+(0.007216×Age×SittingHeight)+(0.02292×(Weight/Height×100))

Afterward, participants were categorized into three groups: pre-PHV (< −1 year from PHV), circa-PHV (±1 year from PHV), and post-PHV (> + 1 year from PHV).

### 2.4 Statistical analysis

Means and standard deviations for participants' characteristics and performance were computed. Data distribution of each variable was assessed using the Shapiro–Wilk test. For variables normally distributed, differences among groups were analyzed using the one-way analysis of variance (ANOVA) with Welch's correction to account for potential heterogeneity of variances. In cases of variables not normally distributed the Kruskal–Wallis test was used. When a significant main effect was found (*p* < 0.05), *post-hoc* comparisons were conducted to identify pairwise differences between maturity status groups. In detail, the Tukey's test was adopted following the one-way ANOVA with Welch's correction and the Dwass-Steel-Critchlow-Fligner (DSCF) pairwise comparisons following the Kruskal–Wallis test. Effect sizes were reported as eta squared (*η*^2^) for the one-way ANOVA, with values interpreted as small (0.01), medium (0.06), and large (≥ 0.14) (30). Cohen's d with 95% confidence intervals (CIs) for each Tuckey *post-hoc* comparison, using the conventional thresholds (small: 0.20; medium: 0.50; large: ≥ 0.80). For non-parametric analyses, epsilon-square (*ε*^2^) was reported as the effect size for the Kruskal–Wallis test, using the same benchmarks for the eta squared (*η*^2^) (31). Rank-biserial correlation (r) with 95% CIs was reported for DSCF pairwise comparisons, with values of 0.10, 0.30, and 0.50 indicating small, medium, and large effects, respectively (30, 32).

All statistical analyses were conducted using Jamovi statistical software [The jamovi project (2025). *jamovi* (Version 2.6) [Computer Software]. Retrieved from https://www.jamovi.org], and the box plots were created with GraphPad Prism (GraphPad Prism version 10.3.1, GraphPad Software, Boston, Massachusetts USA, https://www.graphpad.com).

## 3 Results

Participants' characteristics for the whole sample and for each maturity status group are reported in Tables 1, 2, respectively. All participants were classified into three maturity status groups based on years from PHV (pre-PHV: < −1 year; circa-PHV: −1 to +1 year; post-PHV: > + 1 year). Physical performance outcomes for the whole sample and for each maturity status group are reported in Tables 3, 4, respectively.

**Table 1:** Descriptive statistics of participants' characteristics of the whole sample.

			95% Confidence Interval	
Variable	N	Mean ± SD	Lower	Upper
Age (years)	44	13.12 ± 1.75	12.58	13.65
Height (cm)	44	156.52 ± 12.13	48.70	57.91
Weight (kg)	44	53.30 ± 15.15	152.83	160.21
BMI (kg/m^2^)	44	21.32 ± 3.65	20.21	22.43
APHV	44	13.81 ± 0.53	79.18	83.32
Sitting Height (cm)	44	81.25 ± 6.80	73.28	77.27
Leg Length (cm)	44	75.27 ± 6.55	−1.21	−0.18
Maturity Offset	44	−0.69 ± 1.69	13.65	13.97
Soccer experience (years)	44	6.48 ± 2.36	5.76	7.19

SD, standard deviation; cm, centimeters; s, seconds; kg, kilograms; kg/m^2^, kilograms per square meter; BMI, body mass index; APHV, age at peak height velocity.

**Table 2:** Descriptive statistics of participants' characteristics for each maturity status group.

				95% Confidence Interval	
Variable	Maturity Status	N	Mean ± SD	Lower	Upper
Age (years)	Pre-PHV	19	11.49 ± 0.57	11.21	11.76
	Circa-PHV	15	13.48 ± 0.75	13.06	13.89
	Post-PHV	10	15.67 ± 0.29	15.46	15.88
Weight (kg)	Pre-PHV	19	41.22 ± 10.15	36.32	46.11
	Circa-PHV	15	57.69 ± 8.83	52.80	62.58
	Post-PHV	10	69.67 ± 11.44	61.49	77.85
Height (cm)	Pre-PHV	19	146.05 ± 7.28	142.54	149.56
	Circa-PHV	15	160.13 ± 5.71	156.97	163.29
	Post-PHV	10	171.00 ± 7.79	165.43	176.57
BMI (kg/m2)	Pre-PHV	19	19.13 ± 3.25	17.56	20.79
	Circa-PHV	15	22.46 ± 2.89	20.86	24.06
	Post-PHV	10	23.77 ± 3.25	21.45	26.10
APHV	Pre-PHV	19	13.74 ± 0.44	13.53	13.95
	Circa-PHV	15	13.85 ± 0.57	13.54	14.17
	Post-PHV	10	13.88 ± 0.65	13.41	14.35
Sitting Height (cm)	Pre-PHV	19	75.37 ± 3.02	73.91	76.83
	Circa-PHV	15	82.60 ± 3.72	80.54	84.66
	Post-PHV	10	90.40 ± 3.44	87.94	92.86
Leg Length (cm)	Pre-PHV	19	70.68 ± 5.29	68.13	73.23
	Circa-PHV	15	77.53 ± 4.10	75.26	79.81
	Post-PHV	10	80.60 ± 6.20	76.16	85.04
Maturity Offset	Pre-PHV	19	−2.25 ± 0.53	−2.51	−1.99
	Circa-PHV	15	−0.38 ± 0.64	−0.73	−0.03
	Post-PHV	10	1.79 ± 0.49	1.44	2.14
Soccer Experience (years)	Pre-PHV	19	5.11 ± 1.73	4.27	5.94
	Circa-PHV	15	7.60 ± 1.55	6.74	8.46
	Post-PHV	10	7.40 ± 3.13	5.16	9.64

SD, standard deviation; cm, centimeters; s, seconds; kg, kilograms; kg/m^2^, kilograms per square meter; BMI, body mass index; APHV, age at peak height velocity.

**Table 3:** Descriptive statistics of participants' physical performance outcomes for the whole sample.

			Shapiro–Wilk test	
Variable	N	Mean ± SD	W	*p*
CMJ Height (cm)	44	25.79 ± 5.88	0.982	0.724
Sprint 10 m (s)	44	2.22 ± 0.14	0.976	0.475
Sprint 30 m (s)	44	5.28 ± 0.47	0.962	0.156
V02max (mL/kg/min)	44	41.44 ± 4.27	0.653	<.001
Lower Limb Peak Power (W)	44	1,924.86 ± 873.40	0.947	0.043

N, number, SD; standard deviation; CMJ, countermovement jump; m, meters; cm, centimeters; s, seconds; mL/kg/min, milliliters per kg of body weight per minute; V02max, maximal oxygen uptake; W, watts.

**Table 4:** Descriptive statistics of participants' physical performance outcomes for each maturity status group.

				95% Confidence Interval	
Variable	Maturity Status	N	Mean ± SD	Lower	Upper
CMJ Height (cm)	Pre-PHV	19	22.08 ± 3.92	20.20	23.97
	Circa-PHV	15	26.71 ± 5.29	23.78	29.64
	Post-PHV	10	31.44 ± 4.99	27.87	35.01
Sprint 10 m (s)	Pre-PHV	19	2.31 ± 0.11	2.26	2.36
	Circa-PHV	15	2.21 ± 0.10	2.15	2.27
	Post-PHV	10	2.06 ± 0.08	2.01	2.12
Sprint 30 m (s)	Pre-PHV	19	5.61 ± 0.29	5.47	5.75
	Circa-PHV	15	5.30 ± 0.33	5.11	5.48
	Post-PHV	10	4.65 ± 0.23	4.49	4.81
V0_2_max (mL/kg/min)	Pre-PHV	19	39.46 ± 0.97	38.99	39.93
	Circa-PHV	15	40.27 ± 1.46	39.46	41.09
	Post-PHV	10	46.95 ± 6.14	42.56	51.35
Lower Limb Peak Power (W)	Pre-PHV	19	1,152.83 ± 483.62	919.73	1,385.92
	Circa-PHV	15	2,179.71 ± 486.98	1,910.03	2,449.38
	Post-PHV	10	3,009.46 ± 366.52	2,747.27	3,271.65

N, number, SD, standard deviation, CMJ, countermovement jump; m, meters; cm, centimeters; s, seconds; mL/kg/min, milliliters per kg of body weight per minute; PHV, peak heigh velocity; V0_2_max, maximal oxygen uptake; W, watts.

As shown in Table 3, variables normally distributed were CMJ height and sprint speed tests, whereas VO_2_max and lower limb peak power were not normally distributed.

**Figure 1:**
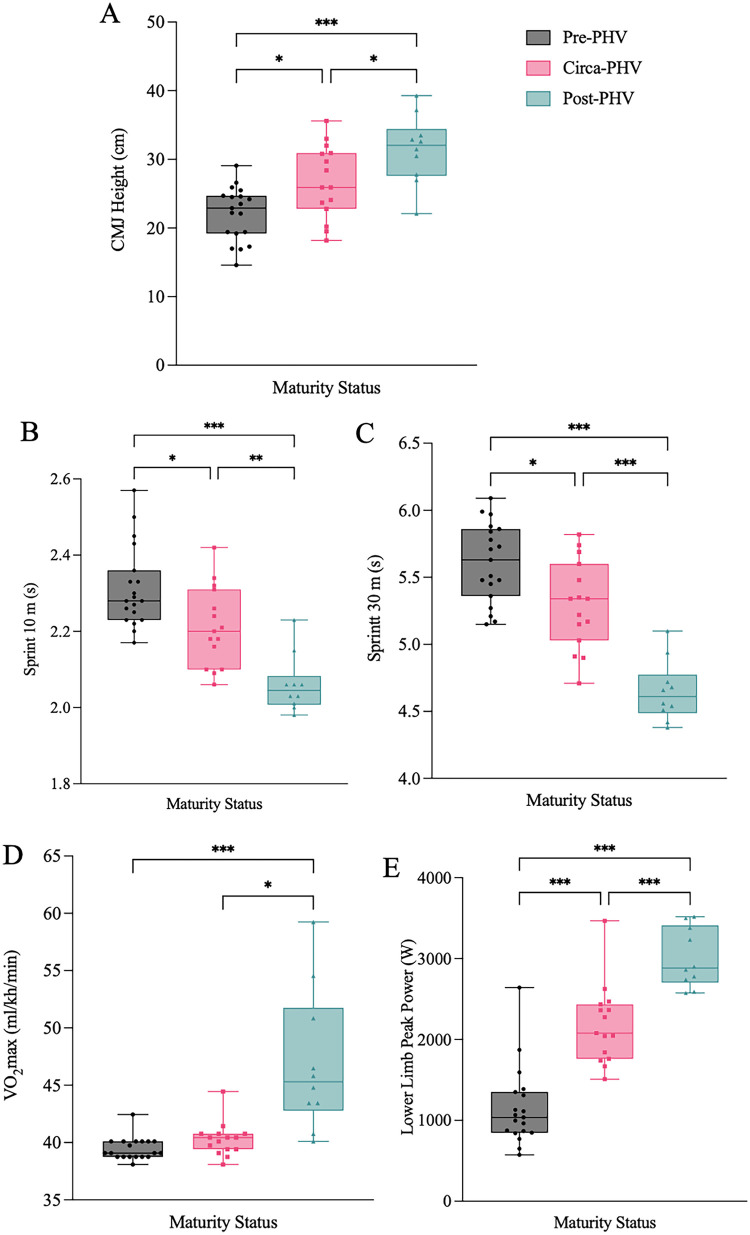
Box plots showing group differences by maturity status (Pre-PHV, circa-PHV, post-PHV) for CMJ height **(A)**, 10-m sprint time **(B)**, 30-m sprint time **(C)**, VO₂max **(D)**, and lower limb peak power **(E)** *p < 0.05; **p < 0.01; ***p < 0.001; PHV, peak height velocity; CMJ, countermovement jump; m, meters; cm, centimeters; s, seconds; mL/kg/min, milliliters per kg of body weight per minute; V0_2_max, maximal oxygen uptake; W, watts.

Statistical analysis revealed significant differences among the maturity status groups for all outcomes. For CMJ height, the results indicated an increase in performance with advancing maturity status (Figure 1A). Participants in the pre-PHV group achieved a mean CMJ height of 22.08 ± 3.92 cm, while those in the circa-PHV group reached 26.71 ± 5.29 cm, and the post-PHV group showed the highest values, that is 31.44 ± 4.99 cm. The one-way ANOVA showed that the differences among groups were statistically significant (*p* < 0.001, *η*^2^ = 0.399). The Tukey's *post-hoc* test confirmed that the post-PHV group jumped significantly higher than both pre-PHV [*p* < 0.001, d = −2.01, 95% CI (−2.91, −1.10)] and circa PHV [*p* = 0.045, d = −1.01, 95% CI (−1.87, −0.16)] groups, while circa-PHV players significantly outperformed their pre-PHV peers [*p* = 0.017, d = −0.99, 95% CI (−1.72, −0.26)].

The same trend was also observed for sprint performance, which increased with increasing maturity status. In detail, in the 10-meter sprint (Figure 1B), the pre-PHV group recorded a mean time of 2.31 ± 0.11 s, compared to 2.21 ± 0.10 s for circa-PHV and 2.06 ± 0.08 s for post-PHV. The group effect was statistically significant (*p* < 0.001, *η*^2^ = 0.507), and the Tukey's *post-hoc* test revealed that the post-PHV group was significantly faster than both pre-PHV [*p* < 0.001, d = 2.53, 95% CI (1.56, 3.50)] and circa-PHV [*p* = 0.002, d = 1.51, 95% CI (0.62, 2.40)] groups, with circa-PHV significantly faster than pre-PHV [*p* = 0.014, d = 1.02, 95% CI (0.29, 1.76)]. Similarly, for the 30-meter sprint (Figure 1C), mean times were 5.61 ± 0.29 s for the pre-PHV, 5.30 ± 0.33 s for circa-PHV, and 4.65 ± 0.23 s for post-PHV players. The difference among groups was statistically significant (*p* < 0.001, *η*^2^ = 0.629) and the *post-hoc* comparisons indicated that post-PHV was significantly faster than both pre-PHV [*p* < 0.001, d = 3.26, 95% CI (2.19, 4.33)] and circa-PHV [*p* < 0.001, d = 2.20, 95% CI (1.24, 3.16)] groups, while the circa-PHV group significantly outperformed the pre-PHV group [*p* = 0.010, d = 1.06, 95% CI (0.32, 1.80)].

As for the aerobic capacity, the VO_2_max showed a similar significant trend by maturity status (Figure 1D). In fact, the pre-PHV had a mean VO_2_max of 39.46 ± 0.97 mL/kg/min, the circa-PHV group 40.27 ± 1.46 mL/kg/min, and the post-PHV group 46.95 ± 6.14 mL/kg/min. The Kruskal–Wallis test indicated a significant group difference (*p* < 0.001, *ε*^2^ = 0.505) and the DSCF pairwise comparisons showed that the post-PHV group had significantly higher VO_2_max than both pre-PHV (*p* < 0.001, rₚᵇ = 0.95) and circa-PHV (*p* = 0.002, rₚᵇ = 0.81) groups, while the difference between pre- and circa-PHV groups did not reach a significant difference (*p* = 0.061, rₚᵇ = 0.45).

Lower limb peak power also showed an association with maturity status (Figure 1E). The pre-PHV group exhibited a mean peak power of 1,152.83 ± 483.62 W, which increased to 2,179.71 ± 486.98 W in the circa-PHV group, and reached 3,009.46 ± 366.52 W in the post-PHV group. The Kruskal–Wallis test confirmed significant differences among the groups (*p* < 0.001, *ε*^2^ = 0.727). The DSCF pairwise comparisons revealed that the post-PHV group had significantly higher peak power than both pre-PHV (*p* < 0.001, rₚᵇ = 0.98) and circa-PHV (*p* < 0.001, rₚᵇ = 0.87) groups, while the circa-PHV group displayed higher peak power than the pre-PHV group (*p* < 0.001, rₚᵇ = 0.86).

## 4 Discussion

This cross-sectional study examined maturity status differences across a multi-domain field-based performance testing battery in youth male soccer players classified by predicted maturity status. Large performance advantages were observed across all tested domains with advancing maturity status. Consistent with the hypotheses, post-PHV players outperformed pre- and circa-PHV players in all performance domains (*p* < 0.001), with effect sizes ranging from large to vary large. These findings extend the existing literature by demonstrating that performance advantages related to maturity status are consistently large across all tested domains. Importantly, while the categorical classification of effect size was uniformly large (all *η*² or *ε*² ≥ 0.40), the magnitude of those effects was domain-specific, ranging from *η*² = 0.40 for CMJ height to *ε*² = 0.73 for lower limb peak power. This domain-specific variation in magnitude effect is a novel contribution of this multi-domain design suggesting that force- and velocity-oriented qualities are sensitive and differ across maturity status groups.

Advanced maturity status was associated with higher vertical jump height and greater lower limb peak power (*p* < 0.001), consistent with previous research demonstrating that maturation influences musculoskeletal architecture and the functional effectiveness of the neuromuscular system (33, 34). The observed effect size were large (*η*^2^ = 0.40 for CMJ; *ε*^2^ = 0.73 for peak power), and the pairwise effect magnitudes are consistent with the well-documented anabolic hormonal environment of post-pubertal maturation (particularly elevated testosterone and growth hormone) which drives skeletal muscle and increases explosive force production (33, 35). The observed performance hierarchy reflects a cascade of physiological, neurological, and morphological changes distinct at each phase of maturation.

During the pre-PHV phase, performance gains are driven by enhanced motor unit recruitment, improved inter-muscular coordination, and increased corticospinal drive rather than hypertrophy (7, 8). Anabolic hormone concentrations remain relatively low, and pre-PHV players represent a sensitive period for neuromuscular coordination and fundamental movement skill development (36).

The circa-PHV phase is characterized by peak growth velocity, during which rapid long-bone elongation can transiently exceed gains in muscle-tendon unit length, disrupting movement patterns and elevating injury vulnerability, the so-called “adolescent awkward phase” (37). This biological difference may partly explain why the circa-PHV group, despite outperforming pre-PHV peers (*p* = 0.02, d = −0.99), demonstrated smaller gains relative to post-PHV players.

Post-PHV players benefit from elevated testosterone and IGF-1, driving skeletal muscle hypertrophy, type II fiber enlargement, and enhanced neuromuscular function (16, 33). Concurrent cardiorespiratory adaptations support the large aerobic gains observed (VO₂max: 46.95 ± 6.14 vs. 39.46 ± 0.97 mL/kg/min in pre-PHV; *ε*² = 0.51). This PHV stage is consistent with the LTAD framework outlined by Duggan et al. (2022) and carry direct implications for S&C programming (36).

Tounsi et al. (2024) examined a large cross-sectional sample of elite youth male soccer players using six fine-grained maturity offset groups (−2.5 to +2.5 YPHV) and reported significant differences in CMJ, sprint, and aerobic performance across groups (38). CMJ values in the groups approximating pre-PHV status (−2.5 YPHV: 23.86 ± 2.63 cm; −1.5 YPHV: 23.82 ± 3.47 cm) and post-PHV status (1.5 YPHV: 32.17 ± 6.03 cm; 2.5 YPHV: 32.25 ± 4.65 cm) are closely in line with those of the present study (pre-PHV: 22.08 ± 3.92 cm; post-PHV: 31.44 ± 4.99 cm). Similarly, in the 30 m sprint performance, the authors found progressive improvements from −2.5 YPHV group (5.05 ± 0.11 s) to 2.5 YPHV group (4.57 ± 0.14 s), as well as in the 10 m sprint performance from 2.07 ± 0.05 s to 1.92 ± 0.08 s between the same maturity ranges, which are consistent with the sprint performance differences observed in the present study between pre- to post-PHV groups (10m: 2.31 ± 0.11 vs. 2.06 ± 0.08 s; 30m: 5.61 ± 0.29 vs. 4.65 ± 0.23 s) (38).

Garcìa-Santamaria et al. (2024) found that post-PHV elite youth soccer players demonstrated higher CMJ performance compared to both mid- and pre-PHV peers (*p* < 0.001), highlighting the role of maturation-related increases in lower limb muscle stiffness as a neuromuscular determinant of jump height (39). In the longitudinal study by Deprez et al. (2015), the authors modelled CMJ development in a large sample of high-level youth soccer players and confirmed that vertical jump performance was higher in earlier-maturing players across observation time points, with fat-free and leg length emerging as key mediators (15). The role of maturation-related hormonal changes in CMJ performance was further underscored by Eskandarifard et al. (2022), who reported that more biologically mature U15 players had significantly higher CMJ values alongside elevated IGF-1 concentrations, reinforcing the endocrine basis of the maturation-explosive power relationship (40). In our study, CMJ height, despite showing a large effect (*η*² = 0.40), represents the smallest difference related to maturity status of the five outcomes assessed, and it suggests that monitoring jump height alone would underestimate the degree of physical disparity between maturity status groups, and that lower limb peak power, sprint speed, and aerobic capacity are more strongly moderated by maturity status.

The large effect sizes observed for lower limb peak power (*ε*² = 0.73) and CMJ height (*η*² = 0.40) are consistent with the meta-analysis by Chen et al. (2024), who reported a pooled mean difference of 3.23 cm (95% CI: 2.32–4.14, *p* < 0.01) for maturation-related differences in explosive leg power in adolescent soccer players, with the largest differences concentrated at the pre-to-post-PHV transition (MD = 4.35 cm, 95% CI: 2.11–6.59, *p* < 0.01) (41). The large group differences in lower limb peak power observed here are plausible given the pronounced hormonal changes during the biological maturation, particularly elevated testosterone and growth hormone concentrations, that accompany advanced pubertal maturation and drive skeletal muscle hypertrophy and concomitant increase in explosive force production (33, 34). Further support for the differences in physical qualities observed in the present study is provided by Synott et al. (2025) who reported significant improvements in the physical bio-motor qualities in elite youth soccer players following an in-season athletic motor skill competency intervention, with maturity status emerging as a key moderating factor of the adaptive response (42). Importantly, it should be noted that body mass is a direct component of the Sayers peak power equation, and the three maturity groups differed substantially in body mass (22). Practitioners and researchers should, therefore, interpret absolute peak power values in the context for substantial body size differences that occur with advancing maturation during adolescence.

A similar pattern was observed for sprint performance, where post-PHV players recorded significant faster 10-m and 30-m times (*p* < 0.001) with large effect size (*η*² = 0.51 and *η*² = 0.63, respectively). These findings are consistent with earlier studies documenting higher sprint performance in more mature youth soccer players, attributed to both greater muscle cross-sectional area and improved neuromotor coordination (33, 35). Tounsi et al. (2021) observed significant differences in 10 and 30 m sprint times across PHV groups in a large sample of male youth soccer players, with post-PHV players consistently recording faster times (10). Garcìa-Santamaria et al. (2024) likewise documented that sprint performance over 10, 20, and 40 m improved progressively across pre-, mid-, and post-PHV stages in elite youth soccer (*p* < 0.001), and that this progression was associated with maturation-related gains in lower limb muscle stiffness (39). Towlson et al. (2018) identified a period of accelerated sprint development between −1.8 and +1.2 years from PHV in a large cohort of soccer players (8–18 years), with a subsequent slowing of developmental gains over time, and this trajectory closely contextualizes the group differences in the present study (26). More recently, Gonzalo-Skok and Bishop (2025) reported that post-PHV elite male footballers demonstrated significantly greater linear sprint performance compared with mid-PHV, supporting the large sprint differences across maturity status groups that we observed (43). Therefore, our finding that 30 m sprint time was the second-largest maturity-related effect size, larger than 10 m sprint, VO_2_max, and CMJ, suggests that maximal velocity sprinting is among the most maturation-sensitive physical qualities in youth male soccer players. This conclusion has a direct implications for talent identification assessment protocols and the weighting assigned to sprint performance in multi-domain physical profiling frameworks.

The aerobic capacity finding warrants particular attention too, as post-PHV players demonstrated markedly higher VO_2_max, with large effect size (*p* < 0.001, *ε*² = 0.51). This is consistent with studies reporting that advanced maturation is associated with increases in cardiac stroke volume, hemoglobin concentration, and muscle mitochondrial density and oxidative enzyme activity, adaptations that are least partially independent of absolute body mass (4, 26, 44, 45). Notably, Mandroukas et al. (2021) demonstrated that relative VO_2_max was significantly higher in youth soccer players compared to untrained peers matched by biological maturation stages, suggesting that maturation-related cardiorespiratory adaptations operate in conjunction with, and at least partially independently of, somatic development (46). Philippaerts et al. (2006) demonstrated in a cohort of youth soccer players that cardiorespiratory endurance and anaerobic capacity showed peak development at PHV, with further progressive gains continuing into the post-PHV period (4). Similarly, Nobari et al. (2022) reported a substantial association between maturity offset and aerobic performance metrics across a competitive soccer season (45). More recently, Berger et al. (2026) demonstrated in a 20-month longitudinal study with young footballers that physical fitness performance aligned more consistently with age at PHV than with chronological age, reinforcing PHV-based classification as an ecologically valid reference framework for aerobic and physical performance profiling (47). The present VO_2_max estimates obtained indirectly via the YYIR1 equation, carry inherent measurement uncertainty (27, 28). Nonetheless, the between-group differences observed is coherent with broader literature on the relationship between maturation and aerobic development in youth soccer (44, 45). Notably, Asimakidis et al. (2022) demonstrated that maturation-related biological adaptations led to significant improvements in sprint and jump performance in 29 youth soccer players even during a 32-week detraining period, underscoring the primacy of maturation-driven biological process in physical development, further validating the use of PHV-based classification for contextualizing performance variation independent of training exposure (48).

Early-maturing players benefit from temporary physiological advantages, including greater absolute strength and aerobic capacity, that confer a competitive advantage during training and match play (49). These advantages extend across the specific physical qualities demanded in soccer, including explosive displacement, maximum sprint speed, and sustained high-intensity running (44, 49). Concurrently, maturation-related physical differences create inequity in talent identification: early-maturing players are over-represented in elite youth development pathways due to their temporary physical superiority, while late-maturing players with substantial latent potential risk being prematurely excluded (50, 51). Evidence suggests that late-maturing players may eventually match or surpass their early-maturing peers in adult performance (52, 53), underscoring the need for talent identification programs that incorporate maturity status alongside physical performance metrics.

The present study strengthens the importance of quantifying the specific magnitude of performance advantages across maturity status groups assessed multi-domain field-based performance testing battery. Prior to this study, the question of which physical domains carry the largest maturation-related disparities within a single cohort could not be answered from within-sample data. The domain-specific effect size hierarchy observed provides practitioners with actionable, rank-ordered guidance: talent identification systems that rely heavily on lower limb power and maximal sprint speed assessments without adjusting for maturity status are at the greatest risk of over-selecting early-maturing players. Conversely, CMJ height, while still showing a large maturity status effect, appears the least confounded of the five domains and may represent a more equitable performance metric in unsupported age-group talent selection contexts.

Advanced biological maturation also influences injury risk. Players with greater muscle mass and strength may be exposed to higher mechanical loads during training and competition, while less mature players competing against physically superior peers face elevated musculoskeletal injury risk (54). The circa-PHV period may represent a particularly vulnerable window. Indeed, rapid bone elongation during PHV can transiently compromise growth plate structural integrity, and attendant disruption of muscle-tendon unit length relationships increases passive musculotendinous stiffness, predisposing players to both acute and overuse injuries (55). Similarly, Johnson et al. (2022) demonstrated in male academy football players that growth-related factors, aside training exposure, were independently associated with increased injury incidence, underscoring the primacy of biological maturation as an injury risk moderator beyond training load (56). These findings reinforce the need for individualized, maturation-sensitive training load management across the full spectrum of maturation development.

The practice of bio-banding, which consist of grouping players according to maturity status rather than chronological age, has shown promise in reducing physical discrepancies and supporting equitable development opportunities (57). Players competing with peers of similar biological development may benefit from improved confidence, engagement, and more appropriate physical and psychological challenges, while coaches gain a clearer view of each player's technical and tactical qualities independently of maturation (58, 59). Incorporating maturation assessments into routine physical profiling is, therefore, essential for objective quantification of physical qualities and for ensuring that late-maturing players are not undervalued during what represents only a temporary development disadvantage (33, 60).

## 5 Conclusions

This cross-sectional study observed performance advantages across all tested physical domains with advancing maturity status. Post-PHV players outperformed pre- and circa-PHV peers in CMJ height, sprint speed, lower limb peak power, and aerobic capacity, with large effect sizes. These findings are consistent with the well-documented physiological adaptations accompanying advanced biological maturation, including hormonal-driven gains in muscle mass, neuromuscular function, and cardiorespiratory capacity. Therefore, maturation is an important contextual factor in youth soccer performance and reinforces the need to account for maturity status when profiling and comparing young athletes. Future studies should employ longitudinal designs and direct maturity assessment methods to further characterize the trajectory and independent contributions of maturation to specific physical performance domains. Future studies should consider longitudinal designs to track individual trajectories.

### 5.1 Practical implications

The large performance differences observed across maturity groups reinforce the importance of incorporating predicted maturity assessments. When profiling young players, as raw performance comparisons within age-group cohorts, may primarily reflect maturational heterogeneity rather than true inter-individual differences in physical capability. Coaches, sports scientists, and talent scouts should integrate maturation-sensitive assessments into routine physical profiling to ensure fair evaluation and reduce selection bias against late-maturing players. Individualized training programs that account for maturity status are recommended to optimize physical development and promote equitable talent identification across the full maturation spectrum.

### 5.2 Strengths and limitations

The cross-sectional design should take casual inference into account, and the small sample size limits generalizability. The subgroup sizes were small and unequal, reducing statistical power. Maturity classification via the modified Mirwald equation carries an inherent prediction error of ± 0.5–1.0 year and known inaccuracies at the extremes of the maturation spectrum (12), potentially introducing group misclassification. A methodological consideration is the link between this Mirwald prediction error and the 1-year classification window widths (pre-PHV: less than −1 year; circa-PHV: −1 to +1 year; post-PHV: more than +1 year). The circa-PHV group is particularly vulnerable, bounded on both sides by thresholds within the error range. VO_2_max was estimated via the YYIR1 equation rather than measured directly (27, 28). Other maturity methods such as the Khamis-Roche could be used even though this estimation carries limitations (e.g., sensitivity to parental height measurement accuracy, lack of parental consent, adopted players). Although we consider only the jump height to measure peak power, it should be noted that the Optojump system provides also flight time (FT) and contact time (CT) parameters, which were not analyzed in the present study and, for future studies, they could be considered providing a more comprehensive neuromuscular performance profile. Moreover, additional sprint distances (e.g., 5 m, 20 m, 40 m) would have provided a more complete characterization of the sprint profile related to maturity status. However, the testing battery does not capture technical and tactical dimensions of soccer performance.

Key strengths include the assessment of multiple physical domains within a single testing battery, enabling direct comparison of effect magnitudes across outcomes. The reporting effect sizes with 95% CIs for comparisons provide a practically informative analysis. Additionally, the use of the modified Mirwald equation, as the maturity status assessment method, represents a genuine applied strength (12). In fact, it requires only standard anthropometric measurements routinely collected in soccer academies, involves no radiation exposure or clinical examination, and has been widely adopted in applied youth soccer research and practice precisely because it offers cost-effective and scalable means of estimating maturity status without the logistical and ethical constrains of gold-standards methods (26).

## Data Availability

The raw data supporting the conclusions of this article will be made available by the authors, without undue reservation.
